# Obstetric Notes and Reflections

**Published:** 1857-05

**Authors:** D. Warren Bricfell

**Affiliations:** Professor of Obstetrics, New Orleans School of Medicine


From New Orleans Med. News and Gazzette.

*Obstetric Notes and Reflections.* By D. Warren Bricfell, M. D, Professor of Obstetrics, New Orleans School of Medicine. A case proving that Menstruation is not only Ovulation, with or without a sanguineous discharge; but that it is, also, the periodical exfoliation of the mucus membrane of the body of the uterus.

About six months ago, I was performing an autopsy in the dead house of the Charity Hospital. On an adjoining table lay the body of a stout young female, who was said to have died of disease of the heart. She had died a few hours previously, and was still quite warm. The thorax and abdomen were laid open. The body had been abandoned, and curiosity led me to examine the internal organs of generation. The uterus and appendages had been cut from the pelvis, and the anterior wall had been laid open. The parts had been thrown aside as possessing no especial interest. The moment I saw the organs I was struck with their being highly engorged with blood, and the uterus was considerably larger than usual. The pelvis was filled with blood which had flowed from the vessels when the organs were detached. The next thing that attracted my attention was the most palpable specimen of *recent* corpus luteum in one ovary. The corpus was large and prominent, and the depression on its centre, exhibiting the point of escape of the ovule, was evident beyond all cavil. In this same ovary one other Graafian vesicle seemed fully matured, the parts surrounding it being highly congested, but the ovule had not escaped. The other ovary was generally congested, but there appeared to be no mature Graafian vesicle.

But the most interesting feature in the case, was the *complete absence of the lining membrane of the cavity of the body of the uterus.* The moment my eye alighted on the inner surface of the organ, I recognized the woodcut of Tyler Smith, in the May, 1856, number of the *Lancet* (Amer. edition,) representing the inner surface of the uterus of a woman who died of apoplexy during the catamenial flow. Nothing could have been more striking than this resemblance; and if I had ever been skeptical in relation to the observations of the author, I was now bound to admit his accuracy. Down *to the os uteri internum* the mucus membrane was gone, and the inner surface of the organ rough, with innumerable blood spots scattered over it. All *below* the os internum was smooth, and in every respect natural in appearance. The difference in sensation conveyed to the finger by touching the two surfaces was as palpable as the impression conveyed to the eye.

The only doubt now remaining about the case was, whether it might not be the uterus which had very recently been delivered of an early ovum. More extended examination, however, proved clearly that this was not the case. The vagina was very small, and its mucus membrane highly corrugated; and there was a well defined hymen. To add to this, the mammæ showed none of the changes generally produced by early pregnancy.

The subject was, to all appearance, about eighteen or twenty years of age, and quite robust. She was the subject of anasarca to a considerable extent, and was said to have died very suddenly—her death being attributed to disease of the heart. I tried to get a more accurate history of her from the nurse of the ward in which she died, but as is too often the case, she only knew that such a woman had been in the ward, had lived, and then had died.

Tyler Smith says, "According to my view, the mucus membrane of the uterus becomes excrementitious every month, and is discharged from the cavity of the uterus in a state of disintegration, and the uterus forms a new mucus coat, by a process similar to the reproduction of lost parts." Coste and others speak of the exfoliation of the mucus membrane of the uterine cavity under certain circumstances; but, so far as I am aware, Tyler Smith is the original advocate of the theory above laid down. After reading all the observations I could procure on this interesting subject, I was altogether inclined to adopt this theory, and the case I have thus described only the more strongly tends to prove its correctness.

The specimen is carefully preserved in the museum of the New Orleans School of Medicine, and is certainly a valuable addition to the already extensive cabinet of rare and interesting specimens from nature.

*Meddlesome Midwifery strikingly illustrated.*—Late in the afternoon of January 8th, 1857, I was called to see Mrs. M. in labor. Found the patient a stout and healthy laboring woman, 26 years of age, and in her first labor. She had arrived at full term and had had no trouble during pregnancy.

Of course I found a midwife in attendance, and from her I gathered the following information. The patient had been taken in labor at 10, P. M., of the 7th, had worked steadily and well along in the first stage, until 10, A. M., of the 8th, when, thinking that she might expedite things somewhat, she ruptured the membranes, notwithstanding she did not think the os uteri was yet sufficiently open to allow the head of the child to pass through. With the evacuation of the waters all urine contractions ceased, and, to use her own language, "labor stopped." She now waited an hour or so, and as the pains did not return, she determined to give some ergot. She gave it in pretty free doses, and soon aroused the organ to a degree of contractile effort "worse than anything they (the friends) had ever seen." This lasted rather more than two hours, when all action again ceased, and up to the late hour at which I saw her, there had been no return. She was merely tormented by "gnawing pains" in the back.

I found her lying on her back, complaining bitterly of her protracted labor, countenance indicative of despondency, and although greatly fatigned and feeling "very weak," still without any *disagreeable* general symptoms. She was glad to see me, but thought it strange that she had not done well in such "experienced hands" as those of her midwife.

The following conversation now ensued between this midwife and myself:

"Can you feel the child?" "Oh, yes." "Is the mouth of the womb entirely open?" "Yes." "What part of the child is presented?" "The head."

I examined the patient per vaginum. Her first answer was correct. She could feel the child readily. The second answer was incorrect. The os uteri was not more than half dilated; indeed, it was rather rigid. The reader may judge how correct the third answer was, when I tell him, that instead of touching the head of the child, my finger passed immediately into the *anus*—the whole vagina being filled with meconium.

Auscultation revealed the fact that the child was still living, though the pulsations of the heart were both feeble and irregular. I waited on her an hour, when, on repeating my examination, I found the os entirely dilatible, but there was nothing approaching uterine action. Suffice it to say, that after waiting as long as I deemed consistent with the safety of the child (having, in the mean time, given ergot freely, but without the effect of arousing the uterus to further expulsive effort,) I passed the hand into the vagina, and hooking the finger in the groin, made traction on the child. This manipulation excited some degree of contraction, and in the course of half an hour the hips were through the vulva. The progress of the child, however, seemed to depend altogether on the traction, the uterus merely following it down. By the time the umbilicus of the child was extruded, I found that life was extinct—there being no pulsation in the cord. The shoulders were slow in rotating and passing through the vulva, and when at last the whole body was extruded, I found that the head, which was very large and well ossified, was detained at the superior strait by the forehead impinging firmly on the linea-ilio-prectinea. The work of remedying this evil, by pushing the occiput up and pulling the forehead down, was quickly done, but the child was still-born.

Finding now that the uterus was contracting imperfectly, and that blood was flowing pretty freely, I passed my hand in and withdrew the placenta. I found it lying loosely in the os uteri, and it came away very readily, its delivery being followed by a free gush of blood. Friction and cold applications over the uterim region, however, brought on a sufficient degree of contraction to place the patient beyond danger. The child was very large indeed. I could not procure scales to weigh it, but I am sure it would have weighed ten pounds.

I have never seen a case more strikingly illustrative of the worse than ignorance of what are usually termed "midwives"—old women who pursue the business of attending their sister women in labor as an actual *trade.* Here is a woman taken in labor with her first child; there being a "breech presentation," the first stage of labor progresses slowly; the midwife (woman of enlarged experience, and consequently very learned in the business,) becoming tired of waiting on the operations of nature, and being perfectly ignorant of the cause of the delay, ruptures the membranes prematurely, and brings all the force of the uterus down on a child which cannot possibly escape, because the os uteri is not yet sufficiently dilated for even the hips to pass through. But after the membranes have been ruptured, there is "an interval of repose; nature has been much fatigued with her efforts in the first stage, and she stops by the wayside to rest. But this is all wrong, says the wise midwife, and I am in a hurry to get home; I will *help* her. Ergot is poured down, and soon nature is aroused from her slumber; and then come uterine contractions "worse than any thing they had ever seen." In two hours the uterus is worn out by ineffectual efforts to expel its contents, the midwife can do no more and the doctor is called in. A few questions put to the midwife not only prove that she has been meddling with the case, but that she is ignorant in the extreme. She not only did not know a rigid and undilated os uteri when she felt it, but absolutely mistook the breech of the child for the head.

